# Ceramide analog [^18^F]F-HPA-12 detects sphingolipid disbalance in the brain of Alzheimer’s disease transgenic mice by functioning as a metabolic probe

**DOI:** 10.1038/s41598-020-76335-4

**Published:** 2020-11-09

**Authors:** Simone M. Crivelli, Daan van Kruining, Qian Luo, Jo A. A. Stevens, Caterina Giovagnoni, Andreas Paulus, Matthias Bauwens, Dusan Berkes, Helga E. de Vries, Monique T. Mulder, Jochen Walter, Etienne Waelkens, Rita Derua, Johannes V. Swinnen, Jonas Dehairs, Felix M. Mottaghy, Mario Losen, Erhard Bieberich, Pilar Martinez-Martinez

**Affiliations:** 1 Department of Psychiatry and Neuropsychology, School for Mental Health and Neuroscience, Maastricht University, Universiteitssingel 50, 6229ER Maastricht, The Netherlands; 2 Department of Physiology, University of Kentucky College of Medicine, Lexington, KY USA; 3 NUTRIM, School for Nutrition and Translational Research in Metabolism, Maastricht University, Maastricht, The Netherlands; 4 Department of Nuclear Medicine and Radiology, MUMC+, Maastricht, The Netherlands; 5 Division of Nuclear Medicine, Uniklinikum Aachen, Aachen, Germany; 6 Department of Organic Chemistry, Slovak University of Technology, Radlinského 9, 81237 Bratislava, Slovak Republic; 7 Department of Molecular Cell Biology and Immunology, Amsterdam Neuroscience, Amsterdam UMC, Amsterdam, The Netherlands; 8 Department of Internal Medicine, Laboratory Vascular Medicine, Erasmus MC University Medical Center, Rotterdam, The Netherlands; 9 Department of Neurology, University Hospital Bonn, University of Bonn, Bonn, Germany; 10 Laboratory of Protein Phosphorylation and Proteomics, KU Leuven, Leuven, Belgium; 11 Laboratory of Lipid Metabolism and Cancer, KU Leuven, Leuven, Belgium; 12 Veterans Affairs Medical Center, Lexington, KY 40502 USA

**Keywords:** Neurodegeneration, Alzheimer's disease, Lipidomics, Nuclear chemistry

## Abstract

The metabolism of ceramides is deregulated in the brain of Alzheimer’s disease (AD) patients and is associated with apolipoprotein (APO) APOE4 and amyloid-β pathology. However, how the ceramide metabolism changes over time in AD, in vivo, remains unknown. Distribution and metabolism of [^18^F]F-HPA-12, a radio-fluorinated version of the ceramide analog N-(3-hydroxy-1-hydroxymethyl-3-phenylpropyl) dodecanamide, was investigated in the brain of AD transgenic mouse models (FAD) on an APOE4 or APOE3 genetic background, by positron emission tomography and by gamma counter. We found that FAD mice displayed a higher uptake of [^18^F]F-HPA-12 in the brain, independently from the APOE4 or APOE3 genetic background. FAD mice could be distinguished from littermate control animals with a sensitivity of 85.7% and a specificity of 87.5%, by gamma counter measurements. Metabolic analysis of [^18^F]F-HPA-12 in the brain suggested that the tracer is degraded less efficiently in the FAD mice. Furthermore, the radioactive signal registered in the hippocampus correlated with an increase of Cer d18:1/20:2 levels measured in the same brain region by mass spectrometry. Our data gives additional proof that ceramide metabolism is different in FAD mice compared to controls. Ceramide analogs like HPA-12 may function as metabolic probes to study ceramide disbalance in the brain.

## Introduction

Evidence correlating Alzheimer’s disease (AD) pathophysiology and altered ceramide metabolism, in the brain, is increasing^1–3^. The global levels of specific ceramide species extracted from middle frontal gyrus, temporal gyrus, inferior parietal lobe, and hippocampus are elevated in AD patients compared to healthy controls^4–6^. Whether these observations pertaining to the pathophysiology of AD are causative or a subsequent event, is still unclear. Nevertheless, recent clinical and preclinical literature points to prodromal changes in ceramide metabolism in AD^7^. Long-chain ceramide species, like Cer d18:1/22:0 or Cer d18:1/24:0, are elevated in plasma before phenoconversion to mild cognitive impairment or AD^8–10^. The levels of ceramide in cerebral spinal fluid correlates with the concentration of some forms of amyloid-β (Aβ), like Aβ1-38 and Aβ1-40, in individuals at high risk of developing AD. This suggests early involvement of ceramide in the pathogenesis of the disease^11,12^. In alignment with these observations, some enzymes responsible for ceramide anabolic or catabolic synthesis are highly expressed and abnormally active in AD brain tissue, compared to healthy controls^13,14^. Aβ peptides activate phosphodiesterases that break down sphingomyelin to produce ceramide in neurons and glial cells^15,16^. The greatest genetic risk factor to develop AD, the apolipoprotein E4 (APOE4), interestingly enough, was associated with increased ceramide levels in the brain compared to APOE3 carriers in AD positive cases^17^. Therefore, the discovery of tools which are designed to measure altered sphingolipid metabolism in the brain, in vivo*,* could be valuable to study ceramide disbalance in pathology and also aid in diagnosing neurodegeneration.

Ceramide transfer proteins (CERTs) are essential proteins of the sphingolipid metabolism^18–21^. Following the synthesis of ceramide in the endoplasmic reticulum (ER), CERTs extract and relocate ceramide to the trans-Golgi where sphingomyelin (SM) is synthesized^22,23^. CERTs have several functional domains involved in this process, including a steroidogenic acute regulatory protein (StAR)-related lipid transfer START domain, that binds to ceramide, a pleckstrin homology domain (PH) that recognizes Golgi phosphatidylinositol 4-monophosphate, and a two phenylalanines in an acidic tract (FFAT) motif that interacts with the ER-resident protein VAMP-associated protein (VAP)^24,25^. When CERTs activity is blocked pharmacologically or compromised genetically by mutating the START domain, SM synthesis is reduced within minutes^26,27^.

The synthetic ceramide analog N-(3-hydroxy-1-hydroxymethyl-3-phenyl propyl) dodecanamide (HPA-12) is a competitive inhibitor of CERTs transport function. HPA-12 displaces endogenous ceramide and occupies the amphiphilic cavity in the START domain of CERTs, preventing ceramide to be shuttled to the Golgi^27,28^. We recently reported the pharmacokinetics and in vivo biodistribution of HPA-12 in wild type animals^29^. We demonstrated that the radiolabel analog, [^18^F]F-HPA-12, enters the central nervous system (CNS) and accumulates, over time in the brain parenchyma without being completely metabolized^29^. However, the possible use of [^18^F]F-HPA-12 in transgenic AD mouse models, was not explored to date. In this study we employed transgenic FAD mice with a mutated amyloid precursor protein (APP)/ presenilin 1 (PSEN1), carrying human APOE4 or APOE3 genes^30^. Littermates not carrying AD mutated genes APP/PSEN1 were used as controls.

In this study, we report that [^18^F]F-HPA-12 accurately differentiates APP/PSEN1 transgenic animals from control animals with a sensitivity of 85.7% and a specificity of 87.5%. This confirms a sound connection between ceramide metabolism and Aβ pathology. However, the tracer did not detect sphingolipid disbalance specifically driven by the APOE4 genes. We propose that differences in radiotracer uptake is due to slower degradation of the tracer in FAD mouse models.

## Results

### Sphingolipid disbalance in FAD (E3FAD, E4FAD) compared to control (APOE3, APOE4) mice

Amyloid pathology progression, neuroinflammation, and behavioral deficits have been partly described before in E3FAD and E4FAD^30–32^. Meanwhile, the impact of APOE4 on sphingolipid metabolism in the brain is still unexplored. Therefore, we addressed the effect of APOE4 background on sphingolipid metabolism by liquid chromatography-electrospray ionization tandem mass spectrometry (LC-ESI/MS/MS) analysis on hippocampal tissue of 8 to 9-month-old female mice.

**Figure 1:**
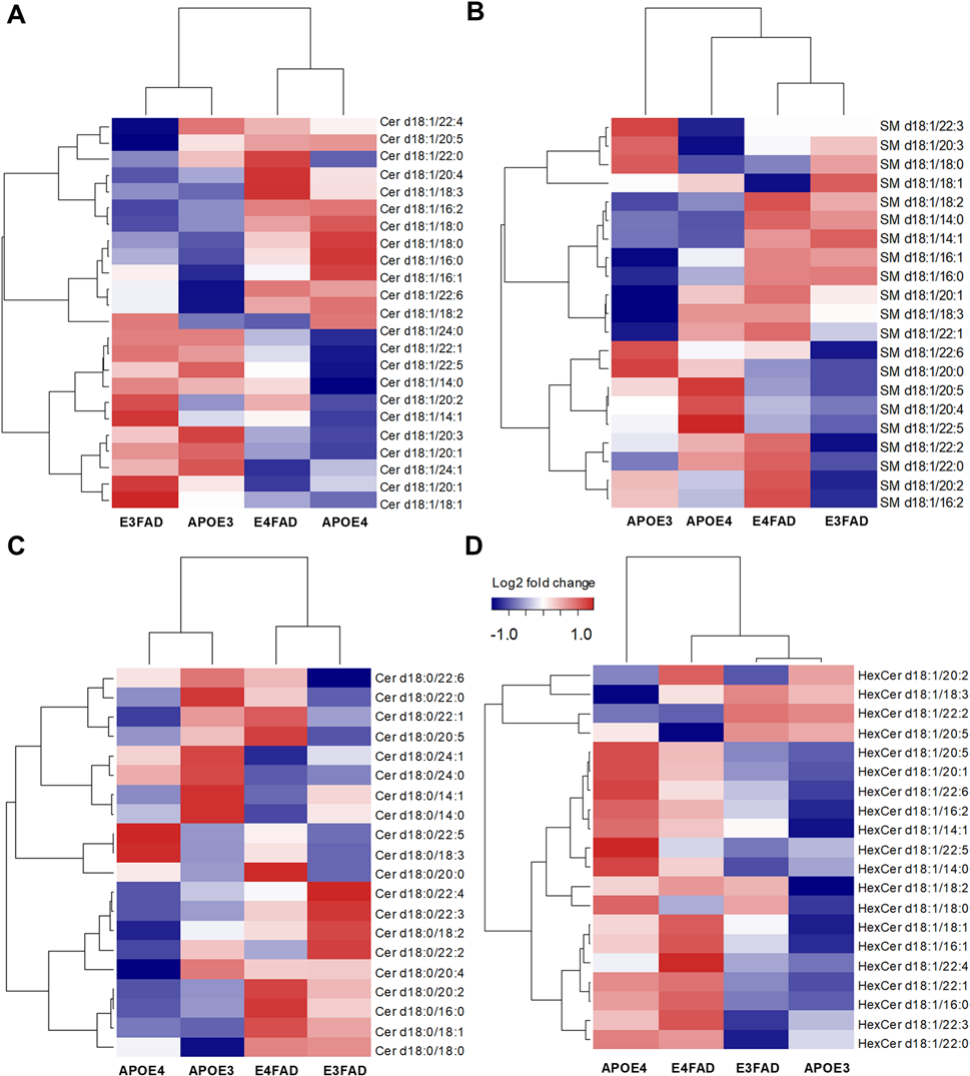
Heatmaps of sphingolipid levels in control (APOE3 and APOE4) and FAD (E3FAD and E4FAD) mice. Ceramide (**A**), Sphingomyelin (**B**), Dihydroceramide (**C**) and Monohexosylceramide (**D**) levels of the hippocampus were measured by LC-ESI/MS/MS. The heat maps show the fold difference (log 2 transformed) of the mean of the relative abundance of each sphingolipid quantified, with a dendrogram showing the hierarchical relationship between the genotypes: APOE3 N = 3, APOE4 N = 6, E3FAD N = 4 and E4FAD N = 5 (data shown as means transformed logarithmically). Within each lipid class, lipid species were hierarchically clustered.

To uncover sphingolipid metabolic differences between APOE3, APOE4, E3FAD, and E4FAD, we performed the first level of analysis by creating a heatmap based on the relative mean quantity of the following sphingolipid classes: ceramides, SM, dihydroceramide, and monohexyl-ceramides (Fig. 1A–D). The hierarchical relationships between genotypes showed that ceramides were mostly different depending on the APOE background. However, the most significant dissimilarities between APOE3 and APOE4 carriers were in the monohexyl-ceramides which were elevated overall in the APOE4 background. Meanwhile, the impact of FAD genes were strong among the dihydroceramide and SM classes. The SM class was mostly reduced depending on specific acyl chain while the dihydroceramide species were overall elevated in FAD mice with some exceptions.

**Figure 2:**
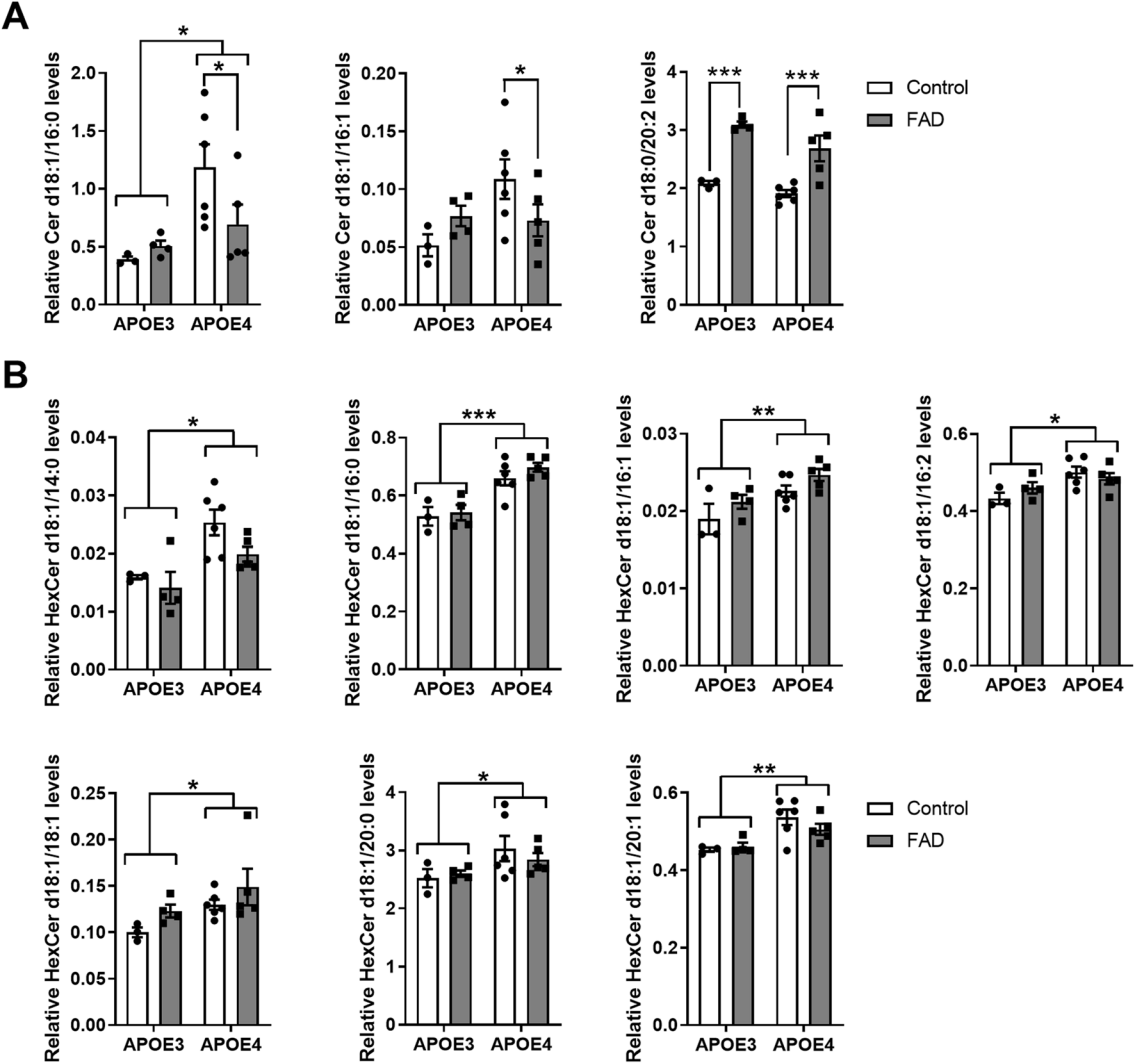
The Cer d18:0/20:2 is increased in FAD mice while Cer d18:1/16:0 and Cer d18:1/16:0 are reduced only in FAD mice carrying APOE4. (**A**) Ceramides were quantified by LC-ESI/MS/MS and the relative abundance of each species was analyzed by MANOVA model with the AD and APOE genes as fixed factors. Among the analyzed ceramides Cer d18:0/20:2 showed the main effect of AD genes while Cer d18:1/16:0 the main effect of APOE genes. Cer d18:1/16:0 and Cer d18:1/16:1 showed a significant interaction effect between AD and APOE genes. (**B**) Monohexosyl-ceramides were quantified by LC-ESI/MS/MS and the relative abundance of each species was analyzed by MANOVA model with the AD and APOE genes as fixed factors. Among the analyzed monohexosyl ceramides HexCer d18:1/14:0, HexCer d18:1/16:0, HexCer d18:1/16:1, HexCer d18:1/16:2, HexCer d18:1/18:1, HexCer d18:1/20:0 and HexCer d18:1/20:1 showed the main effect of APOE genes. Bars represent the mean ± SEM of control (APOE3 N = 3; APOE4 N = 6) and FAD (E3FAD N = 4; E4FAD N = 5) (*p < 0.05, ***p < 0.001).

We further investigated the difference between genotypes by running a MANOVA for each of the 4 classes of sphingolipid with AD and APOE genes as fixed factors, to the same data set. Two sphingolipid classes gave significant MANOVA results for the ceramides and monohexyl-ceramide. The ceramides gave significant main effects of FAD genes (F = 846.210, p = 0.027; Wilks' Lambda = 0.000084) and a statistically significant interaction effect between FAD and APOE genes on the combined dependent variables (F = 368.123, p = 0.0401; Wilks' Lambda = 0.000194). The Cer d18:1/20:2 was significantly elevated in FAD mice (F = 43.821, p = 0.000012). The interaction effects between FAD and APOE genes were confirmed in the following ceramide species Cer d18:0/16:0 and Cer d18:0/16:1 (F = 4.863, p = 0.045 and F = 4.969, p = 0.043 respectively) Fig. 2A. The sphingolipids Cer d18:0/16:0 and Cer d18:0/16:1 were both significantly reduced only in FAD mice with the APOE4 background. The monohexyl-ceramides were exclusively affected by APOE genes (F = 14.657, p = 0.0242; Wilks' Lambda = 0.0167). Seven (HexCer d18:1/14:0, HexCer d18:1/16:0, HexCer d18:1/16:1, HexCer d18:1/16:2, HexCer d18:1/18:1, HexCer d18:1/20:0 and HexCer d18:1/20:1) out of the twenty-two monohexyl-ceramide species analyzed were significantly elevated in the APOE4 background (F = 14.431, p = 0.002; F = 32.898, p = 0.00005; F = 12.2166, p = 0.0036; F = 8.940, p = 0.0097; F = 7.060, p = 0.0187; F = 4.970, p = 0.0432 and F = 14.2356, p = 0.0021 respectively) Fig. 2B.

### Astrocytic ceramide is a driver for Cer d18:1/20:2 elevation in the hippocampus

**Figure 3:**
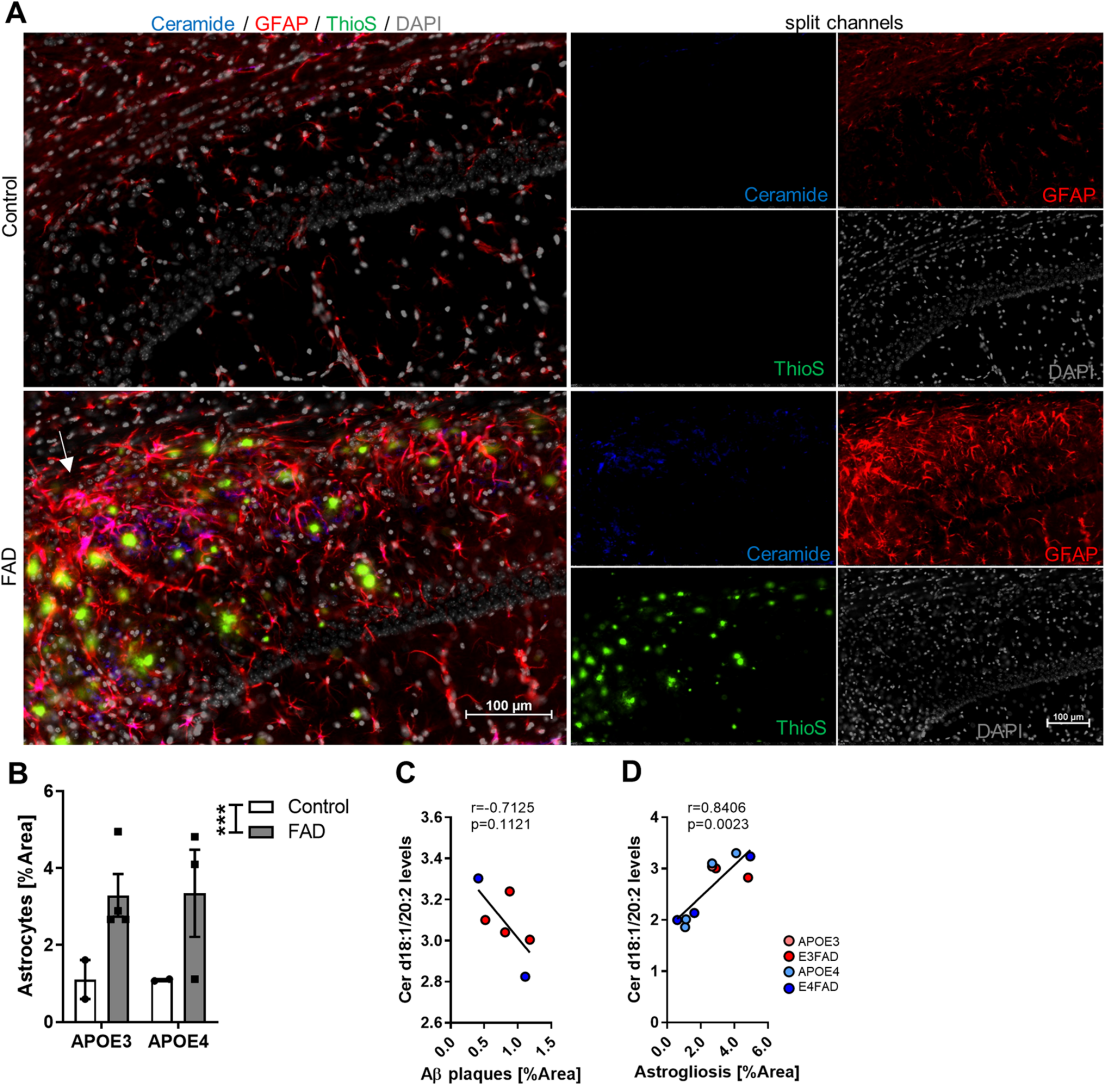
Quantification of hippocampal astrogliosis correlates with Cer d18:0/20:2 levels. (**A**) Representative photomicrographs of nuclei (gray), plaques (green), GFAP (red) and ceramide (blue) fluorescent staining in CA1 region of the hippocampus of control and FAD mice. White arrow points to ceramide and GFAP co-localization (magenta). All photomicrographs were exposed and processed identically. Scale bar 100 µm. (**B**) Immunofluorescent quantification of GFAP measured by the percentage of area. Bars represent the mean ± SEM of control (APOE3 N = 2; APOE4 N = 2) and FAD (E3FAD N = 4; E4FAD N = 3). Student t test ***p < 0.001. (**C**) Correlation between: Cer d18:1/20:2 and plaques quantification expressed as percentage of area in the hippocampus Number of XY Pairs = 6. (**D**) Correlation between: Cer d18:1/20:2 and GFAP quantification expressed as percentage of area in the hippocampus. Number of XY Pairs = 10.

The reason as to why ceramide increases in both the brains of AD patients and AD animal models, is still unknown. However, transgenic animals with severe Aβ pathology show ceramide overproduction, suggesting that the reason is a consequence of aberrant plaques formed in the brain. In addition, the idea that activated astrocytes may contribute to ceramide elevation in the brain of AD patients, has recently been reported^33^. Therefore, we tested these two hypotheses by quantifying plaques with Thioflavin S and astrocytes with anti-GFAP antibody staining in the hippocampus (Fig. 3A,B) and correlated the measurements to Cer d18:1/20:2 levels (Fig. 3C). Co-immunostaining with anti-ceramide antibody indicated high co-localization with GFAP immuno-labeling (Fig. 3A). Quantification of GFAP immunostaining presented a significant increase of astrocytes area in FAD compared to control (Student t test p = 0.0005) (Fig. 3B).

Correlation between the plaques area and Cer d18:1/20:2 levels were not significant (Fig. 3C). Instead, the association between astrocyte staining and Cer d18:1/20:2 levels, was significant (Fig. 3D).

This data suggests that activated astrocytes contribute to Cer d18:1/20:2 elevation in the hippocampus.

### Brain uptake of [^18^F]F-HPA-12 is increased in FAD mice compared to control mice

**Figure 4:**
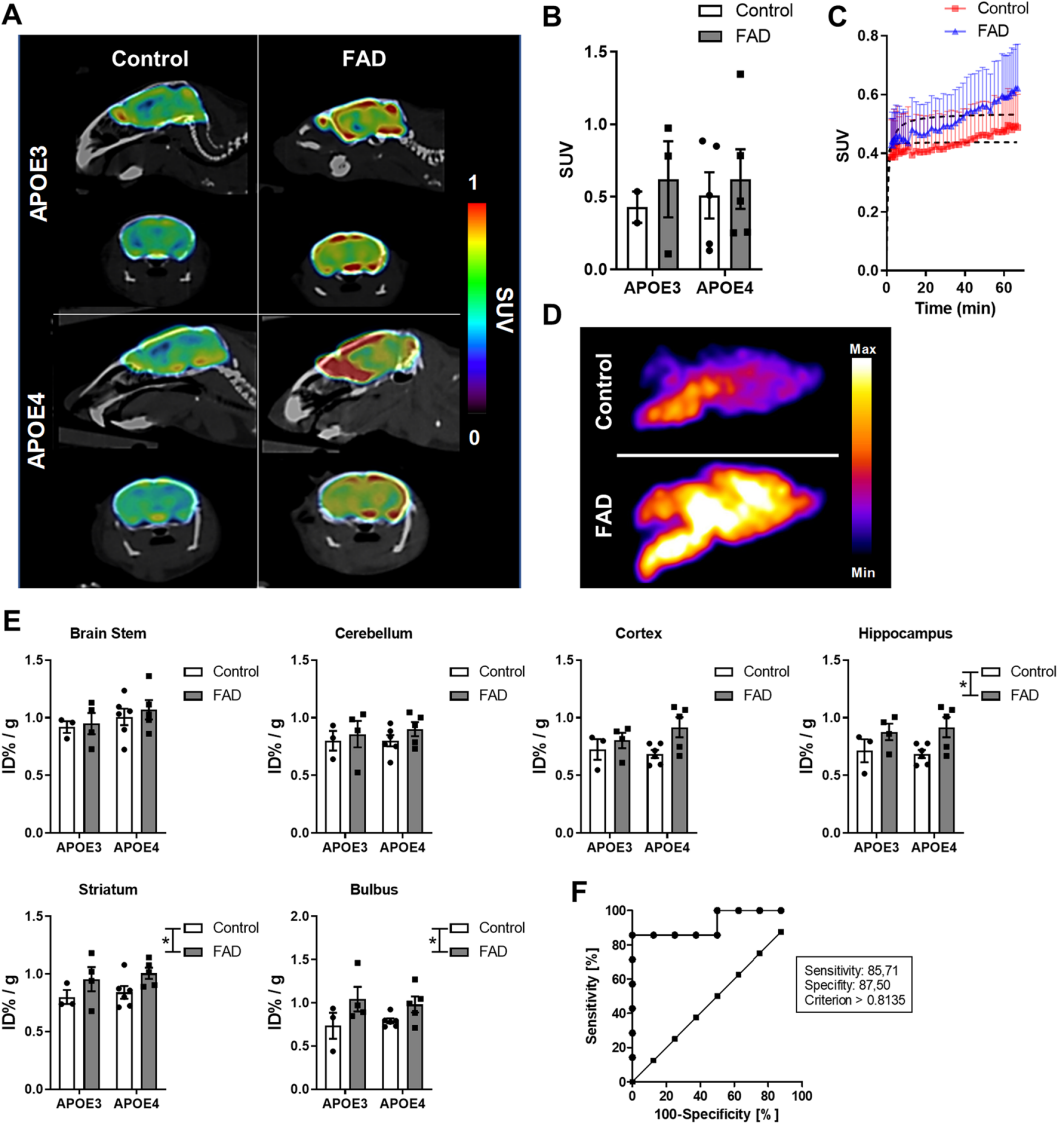
Brain uptake of [^18^F]F-HPA-12 is increased in FAD transgenic animals compared to controls. (**A**) Averaged sagittal and coronal brain slices from 47 to 60 min PET acquisition, co-registered with CT scan of control and AD animals. (**B**) Bar graphs of SUV computed at 60 min of PET acquisition. Bars represent the means ± SEM of data from control (APOE3 N = 3; APOE4 N = 6) and FAD (E3FAD N = 3; E4FAD N = 5). (**C**) Time activity curves (TACs) of control and AD brains expressed as standardized uptake value (SUV). The curves are the means ± S.E.M of data from n = 7–8 mice per experimental group. (**D**) Autoradiograms of control and FAD mice (APOE4 background). (**E**) [^18^F]F-HPA-12 uptake 1.5 h post-injection in the brain stem, cerebellum, cortex, hippocampus, and bulbus. All values were normalized for tissue weight, radioactive decay, and injected dose (ID%/g). Bars represent the means ± SEM of control (APOE3 N = 3; APOE4 N = 6) and FAD (E3FAD N = 4; E4FAD N = 5) (two-way ANOVA, *p < 0.05). (**F**) ROC curve plotted with the true positive rate (Sensitivity %) in function of the false positive rate (100-Specificity %) for different cut-off points (criterion). Each point on the ROC curve (circles) represents a sensitivity/specificity pair corresponding to a decision threshold. In the random classification curve each point is noted with squares.

We previously demonstrated that the radio-fluorinated version of the ceramide analog [^18^F]F-HPA-12, crosses the blood–brain barrier and accumulates in the brain without being completely metabolized. However, we did not explore applications of the radioligand in disease conditions where sphingolipid metabolism is abnormal.[^18^F]F-HPA-12 (injected dose per animal on average 1.8 MBq), was administered into the tail vein, and animals were scanned for 1 h. The PET images displayed an increase in radioactive signal in the brain of FAD animals independently from the APOE4 or APOE3 backgrounds (Fig. 4A). Particularly, the hippocampus uptake was different between the control and FAD (Supplementary Fig. 1A). When analyzing the SUV, computed at 60 min, the FAD genotypes did not show statistically significant differences (Fig. 4B). However, the time-activity curves (TACs) revealed that the accumulation of the radiotracer in the brain parenchyma of the FAD animals was greater than in the control group (Fig. 4C). The comparison between the two curves was carried out comparing the Vmax and Km of the best fit values calculated with the Michaelis–Menten kinetic model (control Vmax = 0.4548 ± 0.0233; AD Vmax = 0.5558 ± 0.0245 and control Km = 0.6015 ± 0.5120; AD Km = 1.197 ± 0.5513; p = 0.0039).

Next, we performed autoradiography and biodistribution to confirm in vivo brain kinetics. 1.5 h post-injection the half brain was sagittally cryosectioned and mounted on glass for autoradiography. This confirmed a higher radio signal in the brain parenchyma of FAD mice brain with different intensities depending on brain region (Fig. 4D). The other brain hemisphere was micro-dissected into the brain stem, cerebellum, cortex, hippocampus, striatum, and bulbus, and analyzed by a gamma counter. The gamma counter measurements confirmed a higher uptake of [^18^F]F-HPA-12 in FAD than in control animals (MANOVA with genotype as independent factor F test = 3.569, Wilks Lambda value = 0.3393 and with p = 0.033) (Fig. 4E). Overall, the hippocampus, striatum and bulbus retained significantly higher levels of the radiotracer (two-way ANOVA respectively F = 7.4164, p = 0.0164; F = 7.4164, p = 0.0396 and F = 6.3690, p = 0.0243).

To determine how accurately [^18^F]F-HPA-12 could distinguish the FAD mice from controls, we ran a Receiver Operating Characteristic (ROC) curve analysis with hippocampus biodistribution expressed in ID% / g (Area: 0.9286; Standard deviation: ± 0.0745; 95% confidence interval: 0.7824 to 1.075; **p = 0,0055)^34^. The tracer with a cut off > 0.8135 had a sensitivity (the ability of the tracer to identify FAD mice correctly) of 85.7% and a specificity (the ability of the tracer to identify control animals correctly) of 87.5% (Fig. 4F). The positive likelihood ratio (+ LR = 6.86) was greater than 1 indicating that the radiotracer brain uptake is associated with AD pathology.

### [^18^F]F-HPA-12 peripheral organ biodistribution

**Figure 5:**
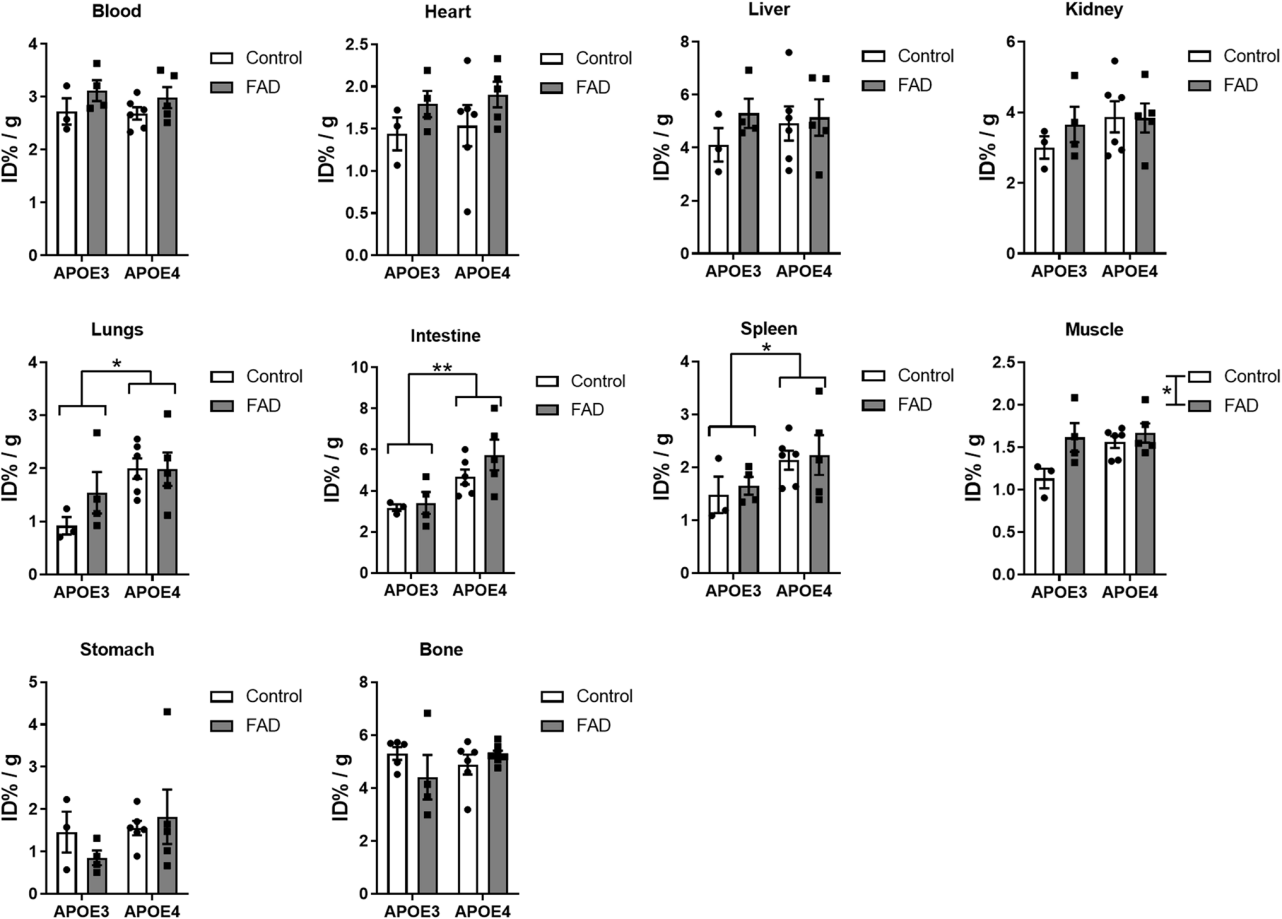
[^18^F]F-HPA-12 biodistribution analysis of peripheral organs.[^18^F]F-HPA-12 organ uptake 1.5 h post-injection in blood, heart, liver, kidney, lungs, intestine, spleen, muscle, stomach, and bone. All values were normalized for tissue weight, radioactive decay and injected dose (ID% / g). Bars represent the means ± SEM of control (APOE3 N = 3; APOE4 N = 6) and FAD (E3FAD N = 4; E4FAD N = 5) (two-way ANOVA, *p < 0.05; **p < 0.01).

[^18^F]F-HPA-12 uptake was studied in the following organs: blood, heart, liver, kidneys, lungs, intestine, spleen, muscle, stomach, and bone (Fig. 5). No significant differences were observed after MANOVA testing combining all the peripheral organ analyzed. However, the univariates analysis for lungs, intestine, and spleen had different uptake depending on APOE background. In all 3 of these organs, APOE4 carriers had larger uptake of the tracer (two-ANOVA respectively F = 6.7306, p = 0.0212; F = 11.7119, p = 0.004 and F = 4.6099 p = 0.0498). Only the muscle among peripheral organs showed a main effect of AD genes (F = 6.2391, p = 0.0255).

### [^18^F]F-HPA-12 is metabolized differently in control than in FAD animals and correlates with Cer d18:1/20:2 levels but not with CERT levels in the hippocampus

**Figure 6:**
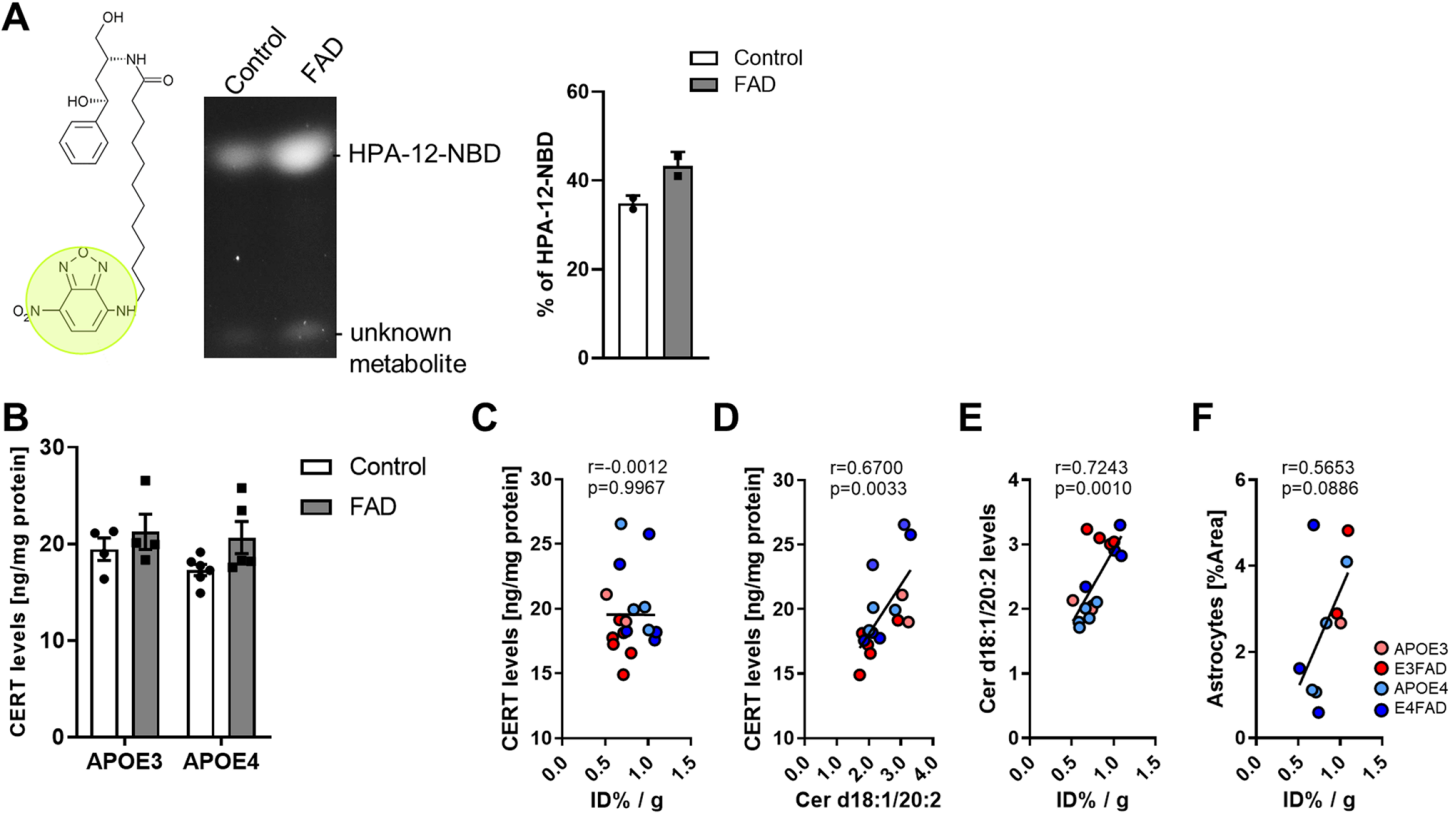
[^18^F]F-HPA-12 radioactivity correlates with Cer d18:1/20:2 levels but not with CERTs concentration in the hippocampus. (**A**) Fluorescent HPA-12 (HPA-12-NBD) chemical structure. Image was drawn using BKChem Wiki 0.13.0 (URL: https://bkchem.zirael.org/wiki/doku.php?id=available_packages&rev=1271765119) Visualization of HPA-12-NBD in hippocampal lipid extract, separated by TLC. Bar graph represent quantification in percentage of intact HPA-12-NBD of 2 controls and 2 FAD brains. (**B**) CERTs measured in hippocampal protein extract by ELISA. Bars represent the means ± S.E.M of control (APOE3 N = 4; APOE4 N = 6) and FAD (E3FAD N = 4; E4FAD N = 5). (**C**) Correlation between Cer d18:1/20:2 and CERT concentration quantified by ELISA in the hippocampus. (**D**) Correlation between radioactive counts and CERT concentration quantified by ELISA in the hippocampus. (**E**) Correlation between radioactive counts and Cer d18:1/20:2 levels in the hippocampus measured by LC-ESI/MS/MS. (**F**) Correlation between radioactive counts and astrogliosis measured by immunostaining in the hippocampus. A color code was given too each genotype. All correlation are based on number of XY Pairs = 17.

We performed analytical studies by HPLC to measure differences in [18F]F-HPA-12 metabolism, between control and FAD animals, on urine, blood, and brains. Urine analysis in control mice indicated that 33% of the radioactivity levels were caused by free fluorine-18 (retention time 2–4 min), 66% by unknown metabolites (retention time 12–14 min), and 1% by [^18^F]F-HPA-12 (retention time 16–18 min). In contrast, the percentage of FAD mice were respectively 50%, 45%, and 5%, suggesting that especially unknown metabolites (retention time 12–14 min) are less present in AD animals. The blood analysis did not show differences in the levels of [^18^F]F-HPA-12 and metabolites between control and AD. Control animals displayed the following percentages: 10% of free fluorine-18 and metabolites, 7% of intact [^18^F]F-HPA-12, 33% bound to plasma protein, and 50% associated with blood cells; while percentages of FAD mice were respectively 12, 6, 25 and 57%. In the brain, we found that [^18^F]F-HPA-12 was less degraded in AD brains compared to control (percentage of the intact compound in control = 26% and AD = 36%). Overall, the radio-analytical study suggested that [^18^F]F-HPA-12 is degraded at a slower rate in AD compared to control animals due to the levels of intact [^18^F]F-HPA-12, which were 5 times higher in the urine and 10% higher in the brain. De-fluorination could be excluded given the fact that the radioactive signal of the bones, as measured by the gamma counter, was similar in all groups. Brain metabolism was further investigated using the fluorescent version of HPA-12 (HPA-12-NBD). The fluorescent group (NBD) was introduced in the sphingoid base of the compound, identical to the radionuclide version of HPA-12. Position C-12 is known to have no interference with the compound’s biological properties. One and a half hour post-intravenous injection, the fluorescent compound was extracted using the Folch method from hippocampal tissue and separated from its fluorescent metabolite by silica gel. The calculations of percentages of intact HPA-12-NBD indicated that the compound is found elevated in FAD, confirming the radio-analytical approach (Fig. 6A).

Next, to investigate if the increase of the radioactive signal found in the brain of AD animals was caused by an increase of CERT protein concentration, we quantified the transporter by enzyme-linked immunoassay (ELISA) in protein extract of the hippocampus. The results of the ELISA did not show any significant difference between the two genotypes (Fig. 6B). Moreover, while the concentration of CERTs in the hippocampus correlated with Cer d18:1/20:2 (Fig. 6C), it did not correlate with the radioactivity levels measured in the same region (Fig. 6D). Having said that, the radioactivity in the hippocampus correlated with Cer d18:1/20:2 which was significantly elevated in FAD mice (Fig. 6E) but not with astrogliosis (Fig. 6F).

Our data suggest that [^18^F]F-HPA-12 is degraded faster in normal conditions compared to AD in the brain, and accumulation in the brain of the tracer is associated with Cer d18:1/20:2 levels in the brain tissue.

## Discussion

Here, we report that the [^18^F]F-HPA-12 dynamics, in vivo, is different in FAD mice brains compared to control. The statistical analysis revealed that the [^18^F]F-HPA-12 uptake was faster and higher in the hippocampus, striatum, and bulbus of AD animals. Metabolic analysis of the brain suggested that the higher levels of [^18^F]F-HPA-12 were due to a slower degradation of the tracer in the brain of FAD mice. Furthermore, while the radiotracer did not correlate with CERTs concentration in the hippocampus, it did correlate with Cer d18:1/20:2 levels, which were found to be significantly elevated in AD, in the same brain region.

The evidence associating the disruption of the sphingolipid metabolism to the molecular mechanisms underlying neurodegeneration is growing. A case in point is an increase of ceramide levels which has been described in the brains of AD patients and the brains of transgenic mice displaying early onset of Aβ pathology^3^. The proposal has been made in which ceramide plays a role in neuronal death by sensitizing neurons to Aβ toxicity and/or directly acting as a pro-apoptotic messenger^35,36^. Besides, ceramide elevation might exacerbate neuroinflammation contributing to pathology progression^33^. These multiple indications supported the proposal to modulate sphingolipid metabolism for the treatment of AD. The introduction of Fingolimod, a sphingosine-1-phosphate (S1P) analog known to modulate S1P receptors^37^, for the treatment of multiple sclerosis further propelled the idea that drugs mimicking sphingolipid structure and bioactivity can be used to treat certain brain conditions. Therefore, establishing new methods to study sphingolipid disbalance in the brain could be valuable to better understand the role of ceramide in disease and discover new therapeutic approaches.

It is thought that sphingolipid and other families of lipids like cholesterol are linked to the amyloid pathology via three mechanisms: (i) increasing Aβ production by interfering with proteolytic activity of β-secretase and γ-secretase, (ii) facilitating Aβ aggregation by direct interaction with the lipid and (iii) mediating Aβ toxicity acting as second messengers or contributing to oxidative stress^5,38–40^. Preclinical studies on genetic mouse models of AD suggest that amyloid pathology is accompanied by ceramide disbalance in the brain of these transgenic mice^36,41^. However, as we previously reviewed it depends on genetic background and sex, and there are inconsistencies in results regarding ceramide levels^3^. In this study we employed transgenic FAD mice with mutated APP/PSEN1 carrying human APOE4 or APOE3 replacement genes. In these transgenic models, the sphingolipid metabolome was never studied before.

Bandaru et al. reported that APOE4 is associated with sphingolipid disbalance only in patients with underlying neurological disorders^17^. AD patients carrying APOE4 variants displayed elevation of ceramides d18:1/18:0 and d18:1/24:1 in the gray matter of the middle frontal gyrus (MFG) and of ceramide d18:1/22:0 in the white matter of the MFG when compared to AD patients carrying APOE3. SM d18:1/22:0 and SM d18:1/24:0 levels were reduced in the MFG gray matter while no difference in SM levels were found in the MFG gray matter. In this work, mass spectrometry analysis on the hippocampus, containing both white and gray matter, showed that APOE4 carriers have higher levels of ceramide d18:1/16:0, which is then reduced in FAD mice. Notably, we reported a substantial increase across almost all monohexyl-ceramide species analyzed in the mice with APOE4 background compared to APOE3 background independently of being FAD mice. This observation could be related to the different glucose metabolism that has been reported in these transgenic animals^42^. Nevertheless, the biggest difference in ceramide composition was found between carriers or non-carriers of APP/PSEN1 mutations. Statistical analysis considering APOE and AD genes as independent factors highlighted APP/PSEN1 genes to be the only independent factor to impact ceramides levels significantly and, in particular, Cer d18:1/20:2. Ceramide increases in the brains of AD patients and AD animal models and the reason as to why is still unbeknownst to us. However, the fact that transgenic animals with severe Aβ pathology show ceramide overproduction, suggests that it is a consequence of aberrant plaque formations in the brain. Recently, the contribution of activated astrocytes to ceramide elevation in the brains of AD patients has been reported^33^. Activated astrocytes propagate Aβ in extracellular vesicles and increase the toxicity of the peptide to neurons, as well^35,43^. Here, we observed that activated astrocytes in FAD mice are associated with increased levels of ceramide, and that there is a correlation between densitometric analysis of GFAP immuno-labeling and Cer d18:1/20:2.Surprisingly, the radiotracer did not correlate with CERTs concentration in the hippocampus. Genome-wide gene-expression analysis on amyloid mice models points to a constant reduction of CERTs protein expression after 4 months of age in the hippocampus, which becomes more relevant at 18 months of age. In our study, we did not find differences in CERTs protein levels by ELISA in 8–9 months old females. However, CERTs levels correlated with Cer d18:1/20:2 levels. This implies that while the CERTs concentration is not enough to explain the differences in uptake of the radiotracer in the hippocampus, it still has a biological association to Cer d18:1/20:2 elevation in FAD mice. In the future, the generation of CERT knock-out mouse will be helpful to determine if the uptake of the tracer depends on CERT levels or function. In fact the metabolic changes of the tracer in the brain may depend on the efficiency of CERT transport. Therefore an impaired CERT transport would result in slower [^18^F]F-HPA-12 metabolism.

The muscles were the only other organ that showed a difference in uptake of the radiotracer dependent on the FAD genotype. The idea that AD pathophysiology affects neurons and muscles similarly is not surprising^44^. Mariani et al. showed an increase of oxidative enzyme activity in muscles of AD patients. Other studies reported that AD hallmarks like Aβ plaques and tau fibrils could be found in the muscle linking peripheral muscle degeneration to neuronal pathology in the brain of AD patients^44–46^. The high uptake of [^18^F]F-HPA-12 in the muscle strengthens the idea that the tracer specifically detects AD pathology. Nevertheless, we do not know if higher uptake in the muscles is caused by an increase in ceramide levels.

Even though [^18^F]F-HPA-12 was able to accurately differentiate between FAD mice and the control with a sensitivity of 85.7% and a specificity of 87.5%, proposing [^18^F]F-HPA-12 as a diagnostic tool in AD is still premature. The study has some limitations. The first limitation is the lipophilic nature of the radiotracer which remains a limiting factor, due to the incredibly slow clearance from the brain and the possibility of high unspecific binding. One hour post-injection the plateau of the slope for [^18^F]F-HPA-12 uptake in the brain, is still not reached. An analog tracer with similar properties but with faster uptake and brain clearance, would be desirable. There are other ceramide analogs with more hydrophilic nature that could be used for this purpose^28^. Secondly, the animals were tested when the Aβ pathology is exceptionally severe and blood–brain barrier damage (BBB) could have occurred. The FAD mice might show BBB damage after 9 months^47,48^. The APOE4 genotype is sufficient to cause damage to BBB vasculature and function, potentially before the age of 12 months, as well^49^. However, we did not observe a difference in uptake between APOE4 carriers and APOE3 suggesting that BBB damage is not affecting [^18^F]F-HPA-12 brain kinetics, in this instance. Also note, the differences in uptake were specific for brain regions like the hippocampus and striatum but not for cerebellum. A study including younger animals should be conducted at the initial stages of the disease. The study should be addressed prior to envisioning the tracer for diagnostic aims. These contributions will help prove a relevant approach. The third limitation is the metabolic analysis which was performed on a limited number of animals, and we did not determine the identity of the HPA-12 metabolites. We were not able to exclude the possibility that the metabolic changes are happening when crossing the BBB and what we are observing is difference in dynamic BBB crossing of the HPA-12, either.

In summary, we pioneered a new method to study sphingolipid metabolism in the brain using a ceramide analog, which can cross the blood–brain barrier and accumulate in the brain. This preliminary study opens a new avenue for compound mimicking sphingolipid structure to study sphingolipid disbalance in the brain and potentially to diagnose diseases by PET.

## Material and methods

### Radiotracer preparation

[^18^F]F-HPA-12 was prepared as previously described^29^. HPLC purification (1.0 mL/min, solvent A; 0.1% TFA in water, solvent B; CH_3_CN) (95% to 0% A in 15 min, 0% A to 25 min) was performed on a Shimadzu UFLC HPLC system equipped with a DGU-20A_5_ degasser, a SPD-M20A UV detector, a LC-20AT pump system, a CBM-20A communication BUS module, and a Scan-RAM radio-TLC/HPLC-detector from LabLogic using an Aeris Widepore column (XB-C18, 3.6 μm, 4.6 mm × 250 mm). A collected fraction was evaporated and re-suspended in vehicle solution PEG400/PBS 1:4 (V/V) previously sterilized by filtration and pH adjusted to 7.4. To dissolve the compound completely, the preparation was warmed up to 37 °C and vortexed vigorously.

### Experimental design and animals

The transgenic mice, E3FAD and E4FAD, were purchased from Dr. Mary Jo LaDu (The University of Illinois; Chicago) and bred in-house as described elsewhere^30^. E3FAD and E4FAD were generated by backcrossing the 5xFAD mice which carries human APP and PSEN1 genes with 5 mutations (APP KM670/671NL (Swedish), APP I716V (Florida), APP V717I (London), PSEN1 M146L (A > C), PSEN1 L286V)^50^ to the APOE-targeted replacement mice, in which the murine APOE gene locus is replaced with the human APOE3 or APOE4 gene. Therefore, E3FAD and E4FAD are homozygous for the APOE3 and APOE4 allele respectively and hemizygous for the APP/PSEN1 genes.[^18^F]HPA-12 was tested on 9–12 month old transgenic female E4FAD and E3FAD, as FAD mice, and littermate non-carriers of mutated APP/PSEN1 genes (referred to as APOE3 and APOE4) as control animals. Animal weight is reported in Supplementary Fig. 1B. All experiments were approved by the Animal Welfare Committee of Maastricht University and followed the laws, rules, and guidelines of the local and national authorities of the Netherlands.

### PET imaging

APOE4, APOE3 (referred to as control), E4FAD and E3FAD (referred to as AD) animals 8–9 months old were anesthetized using a continuous isoflurane (2.5% in O_2_) anesthesia protocol. Body temperature was monitored and maintained using an electrical heating pad during the same time. After intravenous injection of 1.8 MBq of radiotracer [^18^F]F-HPA-12 was administered, animals were positioned supine, and data was acquired, continuously, for 1 h. PET images were acquired using a Siemens MicroPET Focus 120 (Siemens Healthcare) with an axial field of view (FOV) of 7.6 cm and a spatial resolution of about 1.3 mm. The data which was collected was corrected for dead time, random coincidences, and decay. Images were reconstructed using OSEM2D. PET images were analyzed with PMOD 3.7 software. A mouse CT scan was used to define volumes of interest (VOIs) for heart, liver, left kidney and total brain. Time activity curves (TACs) were generated by applying VOIs to the dynamic PET images. TACs were normalized to injected activity and body weight to obtain an outcome measure of standardized uptake value (SUV). Tomographic images were co-registered with the Mirroine mouse T2-MRI template to mask the activity outside the brain. We report the average results of PET slices from 47–60 min acquisition^51,52^.

### [^18^F]F-HPA-12 Biodistribution and metabolism

Biodistribution and metabolism analysis was performed as we previously described^29^. One and half hour post-injection, the mice were terminally anesthetized. Prior to intra-cardial profusion, blood and urine were collected from the mice. Next in the procedure, the brain, skin, eyes, spleen, intestines, liver, stomach, kidneys, lungs, bones, muscles and heart organs were dissected from the animal. The half-brain was further micro-dissected into the brain stem, cerebellum, hippocampus, striatum, cortex, and bulbus. Radioactivity content of all dissected body parts was measured using a 2480 Wizard2 gamma counter (Perkin Elmer). The results were corrected for radioactive decay and expressed as a percentage of total injected dose per gram of tissue (% ID/g).

As previously mentioned, the radio-analytical studies were performed on urine, blood, and brain by HPLC^29^. HPLC elution from 0–1 min, 2–4 min, 12–14 min, and 16–18 min were collected and measured on a gamma counter. At each retention time we expected to elute the following: background radioactivity, free fluorine-18, undetermined metabolite, and intact [^18^F]F-HPA-12, respectively.

### Fluorescent HPA-12 (HPA-12-NDB) brain metabolism

Fluorescent HPA-12 (HPA-12 NDB) was prepared for the first time as described in Supplementary Materials and Methods and Supplementary Figs. 2–5. HPA-12 NDB was used to study HPA-12 brain metabolism. About 6–12.5 µg/animal of HPA-12-NBD was injected IV in 5% ethanol in PBS vehicle (maximum volume injected was of 250 µL/animal). The animals were sacrificed as explained above. The hippocampus was isolated and homogenized in ice cold, high purity water and subjected to the Folch method lipid extraction. In brief, lipids were extracted from 400 µL homogenate by the addition of 1 mL of methanol (CH3OH) followed by the addition of 3 mL of chloroform (CHCl3) and sonicated for 10 min. High purity water (600 µL) was added and the sample sonicated for an additional 10 min. The samples were then centrifuged for 5 min at 2000×*g* and the organic phase was collected and dried under a nitrogen stream. The lipids were dissolved in 50 µL CH3OH and 5 µL extraction was separated by TLC with 95:5 CHCl3:CH3OH. A dilution series of HPA-12-NBD was used as reference for sample migration. The gel was imaged with the Biorad ChemiDOC system. The complete image is reported in Supplementary Fig. 6A.

### Autoradiography

An hour and a half post-injection, the brains were isolated after perfusion, as explained above. The hemibrain was embedded in optimal cutting temperature (OCT) and frozen in liquid nitrogen. The brains were then sectioned in a cryostat with a thickness of 16 µm and mounted-on microscope slides. Once dried, brain sections were incubated in phosphor film overnight. Finally, the film was read on the Typhoon FLA 7000 (Perkin Elmer). Images were processed by applying Gaussian blur and printed identically with ImageJ^53^.

### Brain protein extraction

Brain protein extractions were performed on dissected hippocampus, since a bigger difference in uptake was measured in this brain region. After gamma counter analysis, the hippocampus was powdered with an iron mortar partly emerged in liquid nitrogen and divided into aliquots. One aliquot was further lysed in 0.1% SDS, 0.1% Triton X-100, 1% glycerol, 1 mM EDTA, 1 mM EGTA, PhosSTOP and protein inhibitors (Roche). Total protein content was determined with the Bio-Rad DC (Life Science Group) protein assay following the manufacturer's instructions.

### ELISA for CERT detection

Polyclonal antibodies anti-CERTs used in this assay, polyclonal rabbit 01 (Rb01) and polyclonal rabbit 02 (Rb02) were produced by EUROGENTEC SA Immunization Department based on the purified full-length human protein (recombinant hCERT_L_, 1875 bp NP_005704.1)^54^. Validation of antibodies was performed by coating microplates with 25 ng of recombinant CERTs, produced as previously explained^54^. Then a dilution series (1:900, 2700, 8100, 24,300, 72,900) of the antibodies IgG purified from serum, was applied to the wells. After addition of anti rabbit-biotinylated and streptavidin-HRP (Jackson ImmunoResearch Laboratories Europe Ltd.), signal was developed using 3,3′,5,5′-Tetramethylbenzidine (TMB). Absorption was measured at 450 nm within 30 min of stopping the reaction with 2 M H_2_SO_4_ using the Perkin Elmer 2030 manager system. The detection curve is reported in Supplementary Fig. 6B. For CERT detection in brain samples, microplates were coated with 100 μL of Rb01 diluted to a final concentration of 5 μg/mL using coating buffer (50 mM carbonate/bicarbonate buffer pH 9.6), sealed with a plastic sticker and incubated for 1 h at 37 °C. The standard curve was made with serial dilutions of recombinant CERTs diluted in 10% BSA, 0.02% Tween-20 in PBS (a detection curve is reported in Supplementary Fig. 6C). Biotinylated Rb02 was used as detection antibody diluted to final concentration 5 μg/mL, in 1% BSA and 0.02% Tween-20 in PBS and incubated for 1 h at 37 °C. The Rb02 was biotinylated with an EZ-Link Sulfo-NHS-LC-Biotin kit (ThermoFisher), following company recommendations. Finally, plates were incubated with streptavidin-HRP and developed using TMB. Absorption was measured as stated above.

### Immunofluorescent staining

After perfusion, half-brains were moved sequentially into 4% paraformaldehyde and 30% sucrose before embedding them in OCT and freezing them. The brains were sectioned in cryostat at 30 µm thickness and mounted on microscope glasses and stored in − 20 before immunofluorescent staining. After air drying for 30 min, the sections were rehydrated with PBS and stained with 0.5% Thioflavin S prepared in aqueous solution for 8 min at room temperature. Sections were then washed in 70%, 95% ethanol, distilled water and PBS. Then sections were blocked for 2 h with 3% BSA in PBS and incubated overnight with rabbit anti-ceramide (1:500 dilution)^55^ and mouse anti-Glial fibrillary acidic protein (GFAP) 1:200 (2E1, Santa Cruz). The next day, after 3 step washing in PBS, the sections were incubated with secondary antibodies donkey anti-rabbit Alexa-649 and goat anti-mouse Cy3 for 1 h at room temperature. Pictures were acquired with a fluorescent microscope, Eclipse Ti2-E inverted microscope system (Nikon). Images were processed using Nikon NIS-Elements AR software 5.02.00 equipped particle density analyzer. Graphs and statistics are based on the average of two independent stainings.

### Lipidomics analysis

#### Lipid extraction

Tissues were homogenized with a Precellys 24 in 800 μL of water, of which 700 μL was mixed with 800 μL 1 N HCl:CH3OH 1:8 (v/v), 900 μL CHCl3 and 200 μg/ml of the antioxidant 2,6-di-tert-butyl-4-methylphenol (BHT; Sigma Aldrich). 3 μL of SPLASH LIPIDOMIX Mass Spec Standard (#330707, Avanti Polar Lipids) was added into the extract mix. The organic fraction was evaporated using a Savant Speedvac spd111v (Thermo Fisher Scientific) at room temperature and the remaining lipid pellet was stored at − 20 °C under argon^56^.

#### Mass spectrometry

Mass spectrometry was performed as previously described^56^. Lipid pellets were reconstituted in 100% ethanol. Lipid species were identified and measured by liquid chromatography electrospray ionization tandem mass spectrometry (LC-ESI/MS/MS) on a Nexera X2 UHPLC system (Shimadzu), coupled with hybrid triple quadrupole/linear ion trap mass spectrometer (6500 + QTRAP system; AB SCIEX). Chromatographic separation was performed on an XBridge amide column (150 mm × 4.6 mm, 3.5 μm; Waters) maintained at 35 °C using mobile phase A [1 mM ammonium acetate in water-acetonitrile 5:95 (v/v)] and mobile phase B [1 mM ammonium acetate in water-acetonitrile 50:50 (v/v)] in the following gradient: (0–6 min: 0% B to 6% B; 6–10 min: 6% B to 25% B; 10–11 min: 25% B to 98% B; 11–13 min: 98% B to 100% B; 13–19 min: 100% B; 19–24 min: 0% B) at a flow rate of 0.7 mL/minutes which was increased to 1.5 mL/minutes from 13 min onwards. SM, CE, CER, DCER, HCER, LCER were measured in positive ion mode with a precursor scan of 184.1, 369.4, 264.4, 266.4, 264.4 and 264.4 respectively. TAG, DAG and MAG were measured in positive ion mode with a neutral loss scan for one of the fatty acyl moieties. PC, LPC, PE, LPE, PG, PI, PS were measured in negative ion mode by fatty acyl fragment ions. Lipid quantification was performed by scheduled multiple reactions monitoring (MRM), the transitions being based on the neutral losses or the typical product ions as described above. The instrument parameters were as follows: Curtain Gas = 35 psi; Collision Gas = 8 a.u. (medium); IonSpray Voltage = 5500 V and − 4,500 V; Temperature = 550 °C; Ion Source Gas 1 = 50 psi; Ion Source Gas 2 = 60 psi; Declustering Potential = 60 V and − 80 V; Entrance Potential = 10 V and − 10 V; Collision Cell Exit Potential = 15 V and − 15 V.

The following fatty acyl moieties were taken into account for the lipidomic analysis: 14:0, 14:1, 16:0, 16:1, 16:2, 18:0, 18:1, 18:2, 18:3, 20:0, 20:1, 20:2, 20:3, 20:4, 20:5, 22:0, 22:1, 22:2, 22:4, 22:5 and 22:6 except for TAGs which considered: 16:0, 16:1, 18:0, 18:1, 18:2, 18:3, 20:3, 20:4, 20:5, 22:2, 22:3, 22:4, 22:5, 22:6.

#### Lipidomics data analysis

Peak integration was calculated with the MultiQuant software version 3.0.3. Lipid species signals were corrected for isotopic contributions (calculated with Python Molmass 2019.1.1) and were quantified based on internal standard signals and adheres to the guidelines of the Lipidomics Standards Initiative (LSI) (level 2 type quantification as defined by the LSI).

### Statistical analysis

The statistical analysis was performed using RStudio 1.2.5033^57^, IBM SPSS statistic version 26, or GraphPad Prism version 8.4.3 (686). Matrices of the ceramides, SM, dihydroceramide, and monohexyl-ceramides for the hierarchical cluster analysis and heatmaps were generated with RStudio. The four classes of sphingolipids ceramides, SM, dihydroceramide, and monohexyl-ceramides were further analyzed by multivariate analysis with SPSS with APOE and FAD genes (APP/PSEN1) as independent factors. When Wilks' Lambda test was found significant, MANOVA was followed by a series of ANOVA for each acyl-chain species, and LSD was used for post hoc testing eventually. Biodistribution data were analyzed using two-way ANOVAs, with APOE genes and AD genes as independent factors; the LSD post hoc test was used for multiple comparisons. A p-value of < 0.05 was considered significant. Time activity curves for the brain, liver, heart, kidneys, and lungs were acquired with PMOD2 as previously described^29^, and were tested for statistical differences between AD and littermate controls by comparing the Vmax or Km of the best fit values calculated with the Michaelis–Menten kinetic model. A Pearson or Spearman correlation was used to determine the relationship between radiotracer activity in the brain and CERT relative quantification, and the four sphingolipid species under investigation mentioned above. The diagnostic performance and accuracy of [^18^F]HPA-12 were also evaluated using Receiver Operating Characteristic (ROC) curve analysis^34^.

## Supplementary information

Supplementary Information.

## Data Availability

The datasets used and/or analyzed during the current study are available from the corresponding author on reasonable request.
