# Supplementary material for: Ceramide analog [18F]F-HPA-12 detects sphingolipid disbalance in the brain of Alzheimer’s disease transgenic mice by functioning as a metabolic probe
Source: Sci Rep. 2020 Nov 9;10:19354. doi: 10.1038/s41598-020-76335-4 (PMC7652882; doi:10.1038/s41598-020-76335-4)
Supplement: Supplementary file 1 — Supplementary Information. [file 41598_2020_76335_MOESM1_ESM.pdf]

Supporting Information

Ceramide analog [^{18}F]F-HPA-12 detects sphingolipid disbalance in the brain of Alzheimer's disease transgenic mice by functioning as a metabolic probe

Simone M. Crivelli[1,2], Daan van Kruining[1], Qian Luo[1], Jo Stevens[1], Caterina Giovagnoni[1], Andreas Paulus[3,4,5], Matthias Bauwens[3,4], Dusan Berkes[6], Helga E De Vries[7], Monique T. Mulder[8], Jochen Walter[9], Etienne Waelkens[10], Rita Derua[10], Johannes V Swinnen[11], Jonas Dehairs[11], Felix M. Mottaghy[3,4,5], Mario Losen[1], Erhard Bieberich[2], Pilar Martinez-Martinez[1]*

Supplementary Materials and Methods

^{1}H NMR and ^{13}C NMR spectra were recorded using a Varian 600 MHz spectrometer (600 MHz for ^{1}H and 150.9 MHz for ^{13}C). Chemical shifts (δ) were reported in parts per million (ppm) relative to tetramethylsilane (TMS, δ = 0.00 ppm) and referenced to deuterated solvent signals ($CDCl_3$, δ = 7.26 ppm (^{1}H) and δ = 77.16 ppm (^{13}C)). Analytical thin-layer chromatography (TLC) was carried out on silica gel glass plates (60 F254) using UV light (λ = 254 nm and λ = 366 nm) to visualize the compounds. Purifications using flash chromatography were performed on silica gel (40–73 µm). FT-IR-spectra were recorded with a diamond ATR unit. The melting point was determined in an open capillary tube and is uncorrected. Optical rotations were measured on a JASCO P-1020 or POLAR L-mP (IBZ Mestechnik) polarimeter (concentration, c, is given as g/100 mL). High resolution mass spectra (HRMS) were recorded on Orbitrap Velos PRO, Thermo Scientific 5 machine. Commercially available reagents purchased as reagent grade, tetrahydrofuran (THF) (extra dry), and diisopropylethylamine (DIPEA) were used as received. 1-Phenyl-3-amino-butane-1,4-diol (**A**) was prepared by our method. [1] Starting NBD labeled active ester - 2,5-dioxopyrrolidin-1-yl 12-(7-nitrobenzo[c][1,2,5]oxadiazol-4-ylamino) dodecanoate (**B**) was prepared as previously described [2].

Experimental Procedure

N-[(2R,4S)-1,4-dihydroxy-4-phenylbutan-2-yl]-2-[(7-nitro-benzo[c][1,2,5]oxadiazol-4-yl)amino]dodecanamide (HPA-12-NBD)

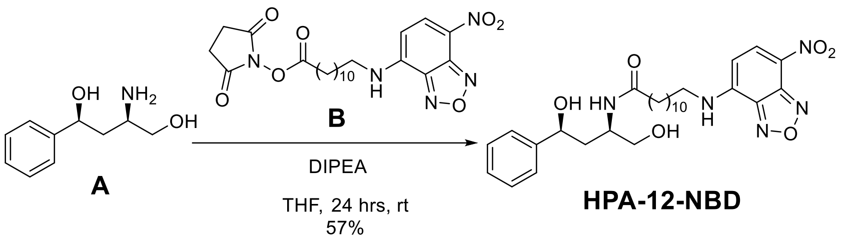

To the solution of **A** (1-phenyl-3-amino-butane-1,4-diol, 156 mg, 0.861 mmol) and DIPEA (111 mg, 0.861 mmol) in THF (10 mL) the active ester of NBD-dodecanoic acid (**B**) (2,5-Dioxopyrrolidin-1-yl 12-(7-nitrobenzo[c][1,2,5]oxadiazol-4-ylamino)dodecanoate, 409 mg, 0.861 mmol) was added in one portion. The reaction mixture was stirred at rt for 24 hrs, concentrated under vacuum and separated on silica gel, eluting with dichloromethane : methanol mixture 100:0→50:1→20:1. The **HPA-12-NBD** was obtained as a red solid in the yield of 264 mg (57 %). **m.p.** 73-75 °C, $[\alpha]_D^{20}$ = +3.4 (c 0.15, MeOH).

^{1}H-NMR, COSY (600 MHz, $CDCl_3$, TMS): δ 8.48 (d, *J* = 8.6 Hz, 1H, H-6“), 7.36 – 7.23 (m, 5H, Ph), 6.61 (bs, 1H, N<u>H</u>), 6.45 (d, *J* = 6.6 Hz, 1H, N<u>H</u>-CO), 6.17 (d, *J* = 8.6 Hz, 1H, H-5“)), 4.83 (dd, *J* = 9.0, 3.3 Hz, 1H, H-4´), 4.08 (dt, *J* = 6.3, 5.7 Hz, 1H, H-2´), 3.73 – 3.63 (m, 2H, H-1´), 3.54 – 3.43 (m, 2H, H-12), 2.18 (t, *J* = 7.5 Hz, 2H, H-2), 2.05 (ddd, *J* = 14.7, 5.5, 3.3 Hz, 1H, H-3´), 1.95 (ddd, *J* = 14.7, 9.1, 7.0 Hz, 1H, H-3´), 1.79 (p, *J* = 7.3 Hz, 2H, H-11), 1.61 (p, *J* = 7.3 Hz, 2H, H-3), 1.45 (p, *J* = 7.1 Hz, 2H, H-10), 1.41 (p, *J* = 7.2 Hz, 2H, H-9), 1.28 (m, 10H, H-4, H-5, H-6, H-7, H-8).

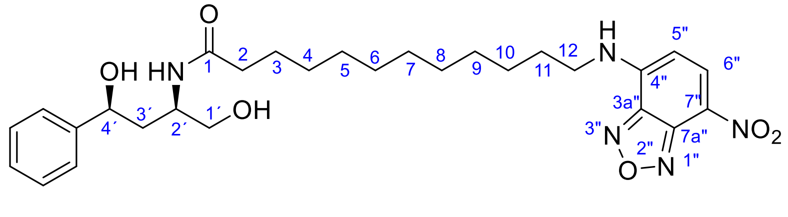

^{13}C-NMR, HSQC, HMBC (150.9 MHz, $CDCl_3$, TMS): δ 174.4 (C-1), 144.3 (*C*q-7a”, Ar), 144.2 (*C*q-3a”, Ar), 144.1 (*C*q-7”, Ar), 144.0 (*C*q-Ph), 136.6 (*C*-6”, Ar), 128.6 (*C*-Ph), 127.8 (*C*-Ph), 125.6 (*C*-

Ph), 123.7 (*C*q-4”, Ar), 98.5 (*C*-5”, Ar), 72.1 (*C*-4´), 65.8 (*C*-1´), 50.7(*C*-2´), 44.0 (*C*-12), 40.7(*C*-3´), 36.8 (*C*-2), 29.2, 29.2, 29.2, 29.1, 29.1, 29.0(*C*-9), 28.4(*C*-11), 26.8 (*C*-10), 25.6 (*C*-3).

IR ATR (cm^{-1}): 3300, 2916,2850, 1639, 1624, 1556, 1264, 1242

HRMS (HESI): m/z $[M+H]^+$ calcd for $C_{28}H_{40}N_5O_6^+$: 542,29731, found: 542,29820; m/z $[M+Na]^+$ calcd for $C_{28}H_{39}N_5O_6Na^+$: 564,27926, found: 564,28002

Supplementary references

1. Santos, C., et al., *The CERT antagonist HPA-12: first practical synthesis and individual binding evaluation of the four stereoisomers.* Bioorg Med Chem, 2015. **23**(9): p. 2004-9.
2. Crivelli, S.M., et al., *Synthesis, Radiosynthesis, and Preliminary in vitro and in vivo Evaluation of the Fluorinated Ceramide Trafficking Inhibitor (HPA-12) for Brain Applications.* J Alzheimers Dis, 2017. **60**(3): p. 783-794.

Supplementary figures

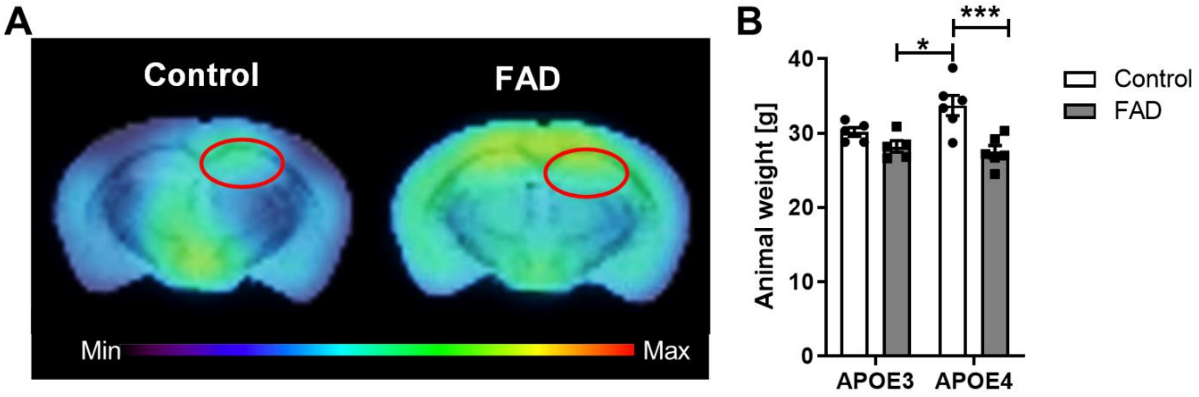

Supplementary Figure 1. FAD animals have lower body mass and higher uptake of [^{18}F]F-HPA-12 in the hippocampus region. A) Averaged coronal brain slices from 0 to 60 minutes PET acquisition of control and FAD mice were overlaid to Mirroine mouse T2-MRI template. Red circle highlights the hippocampus. B) Bar graphs of animal's weight measured the day of the experiments. Bars represent the means ± S.E.M of control (APOE3 N=4; APOE4 N=6) and FAD (E3FAD N=4; E4FAD N=5). (ANOVA main effect of FAD genes $F = 17.07$, $p=0.0006$; interaction between APOE and FAD genes, $F = 4.711$, $p=0.0436$; Bonferroni post-hoc test *$p<0.05$, ***$p<0.001$).

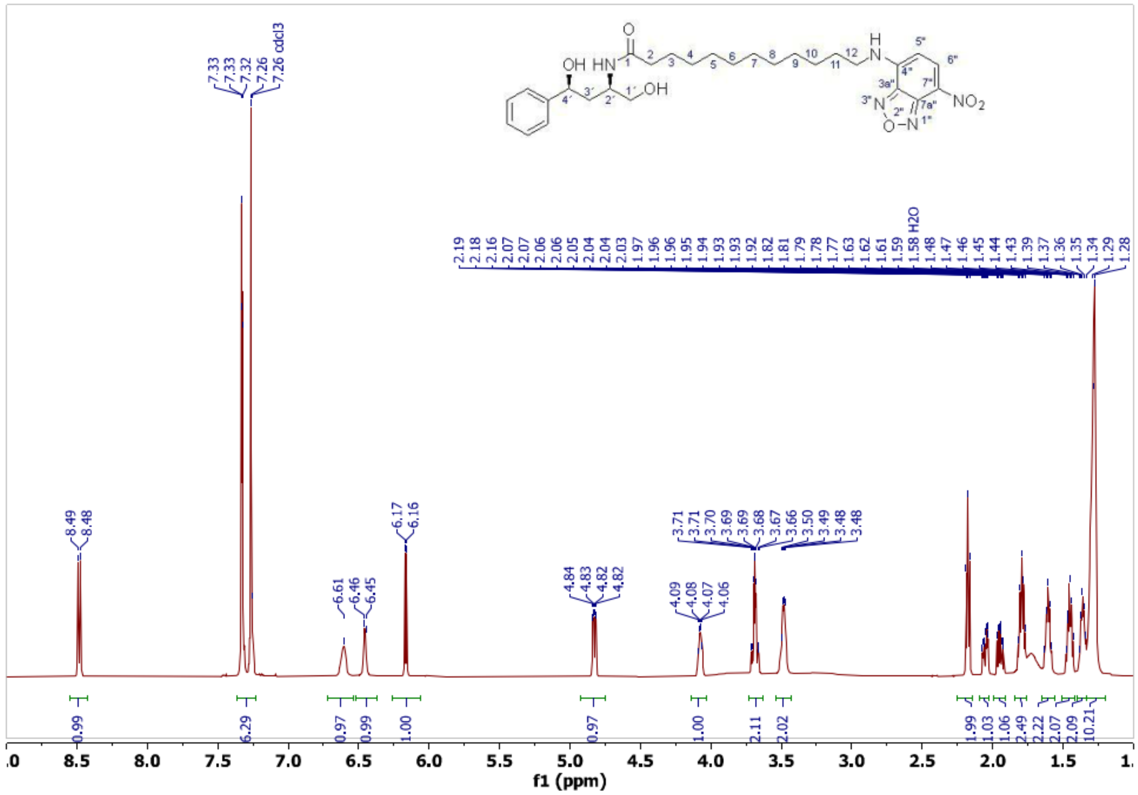

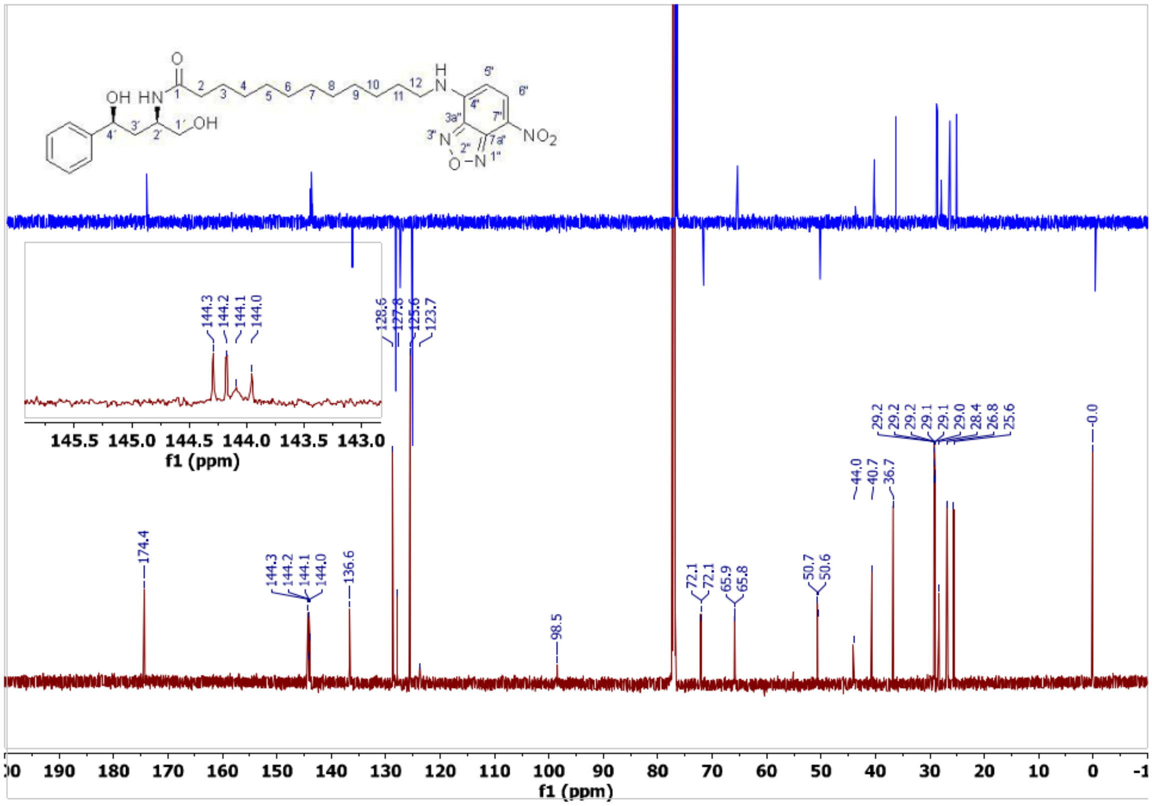

Supplementary Figure 2. HPA-12-NBD ^{1}H and ^{13}C NMR, APT (600 MHz, $CDCl_3$)

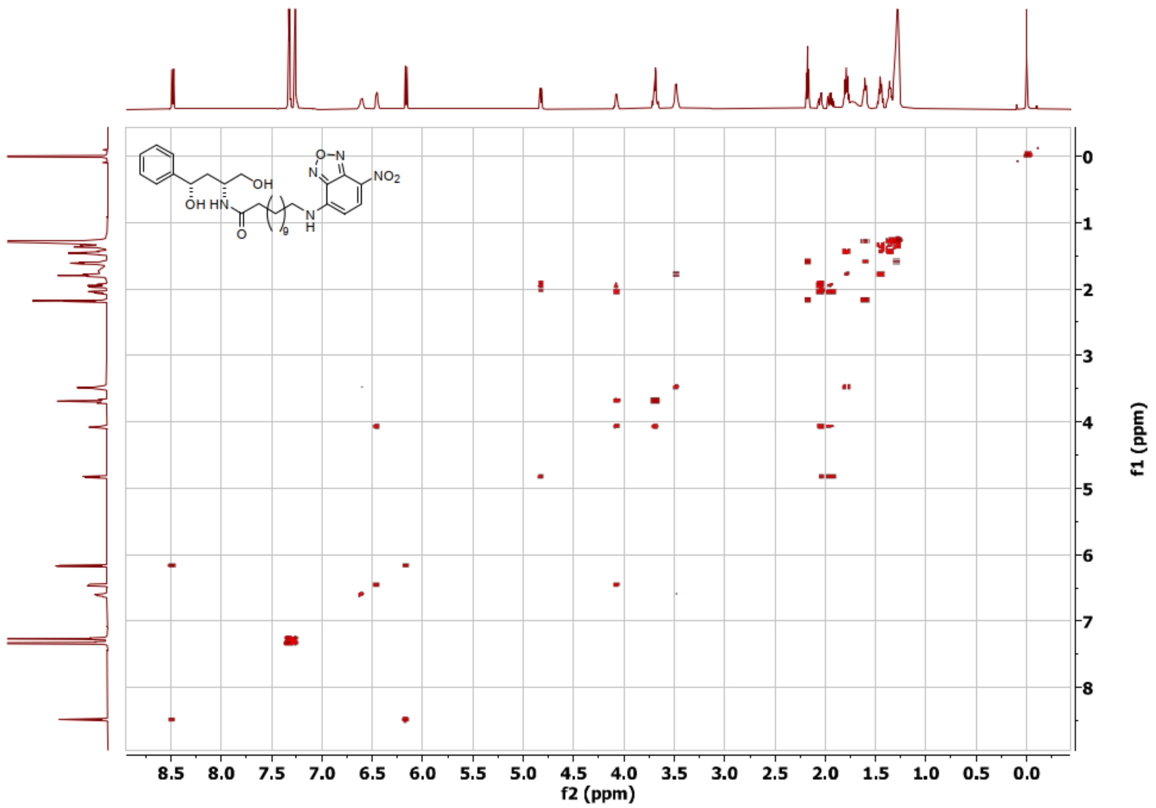

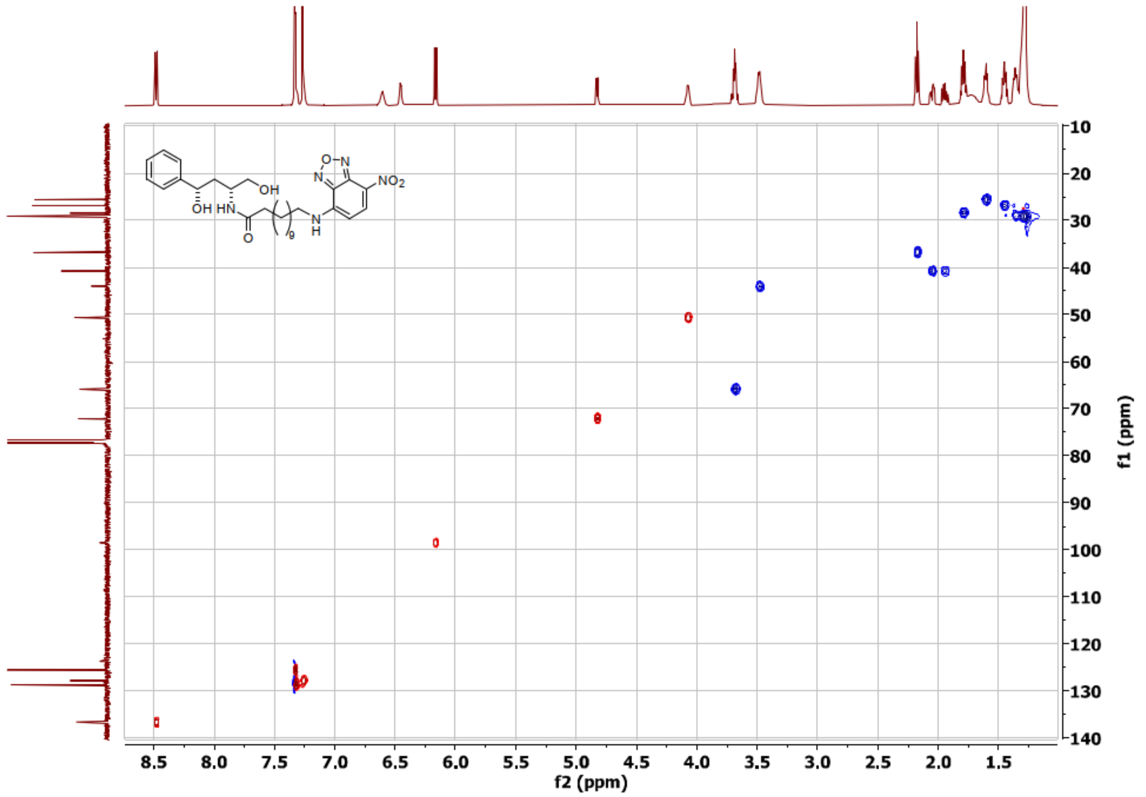

Supplementary Figure 3. HPA-12-NBD COSY and HMBC spectra (600 MHz, $CDCl_3$)

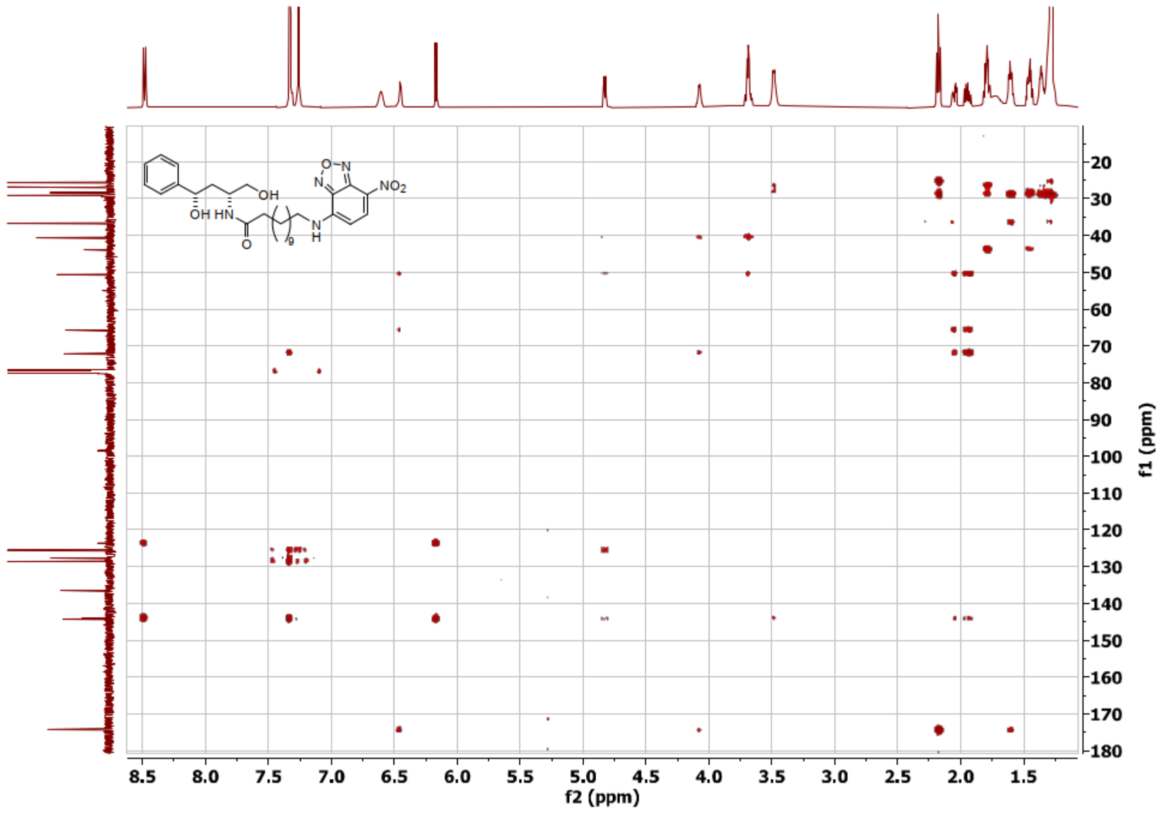

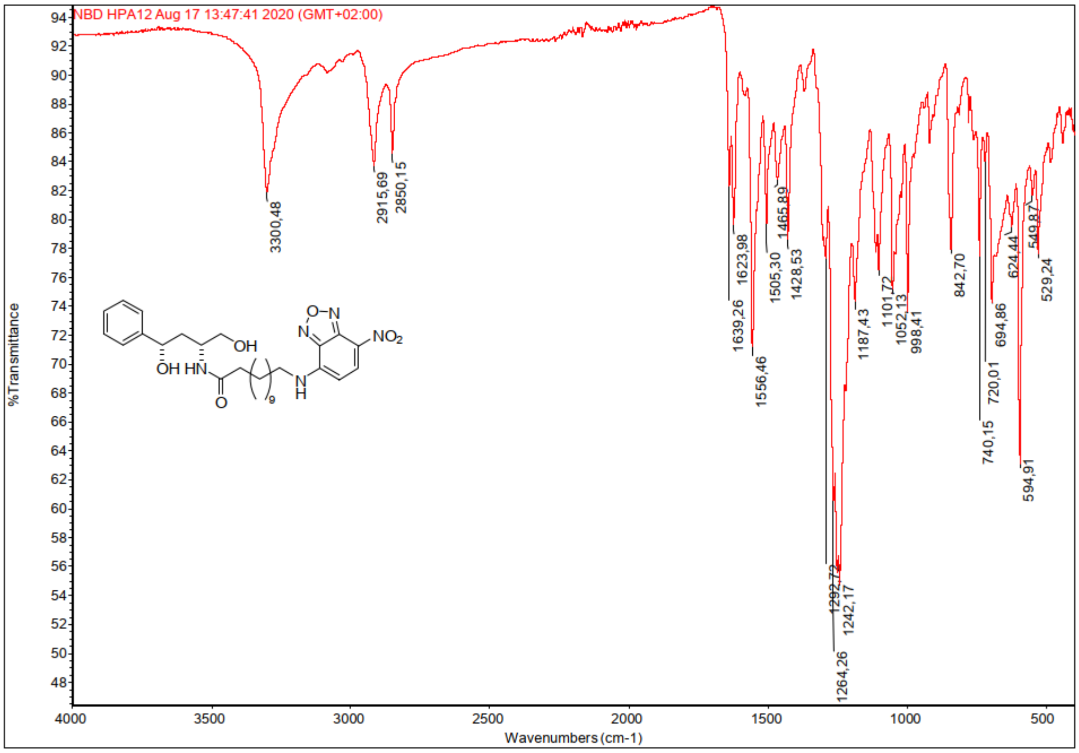

Supplementary Figure 4. HPA-12-NBD HSQC (600 MHz, $CDCl_3$) and IR ATR spectra

HPA-12-NBD

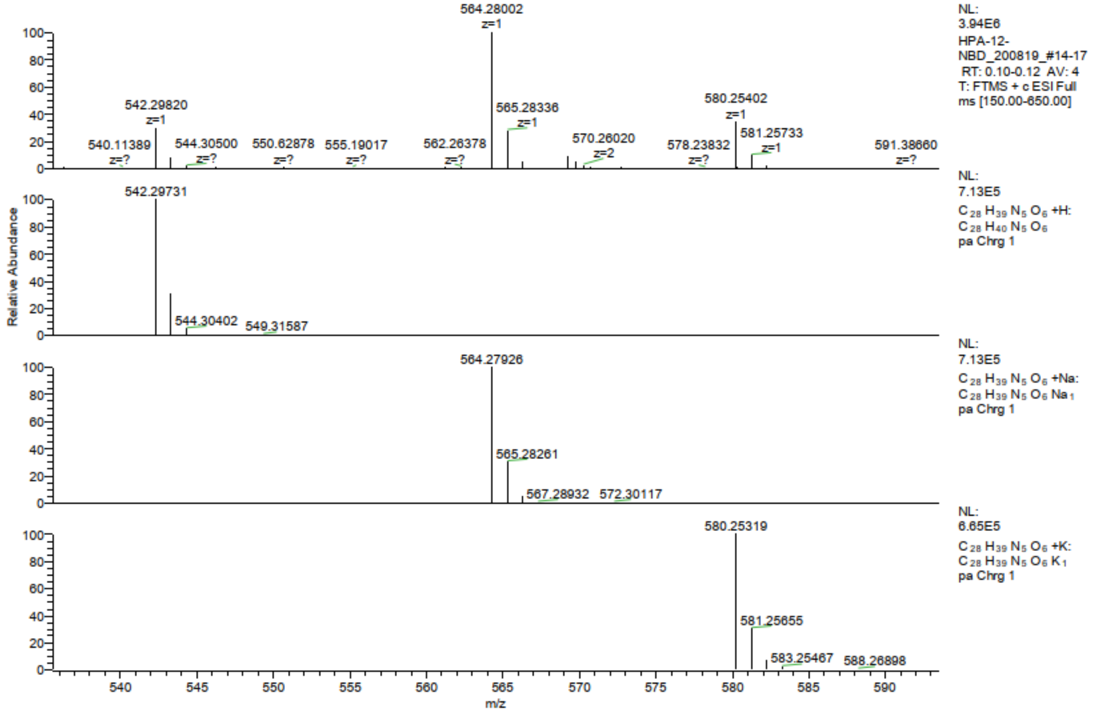

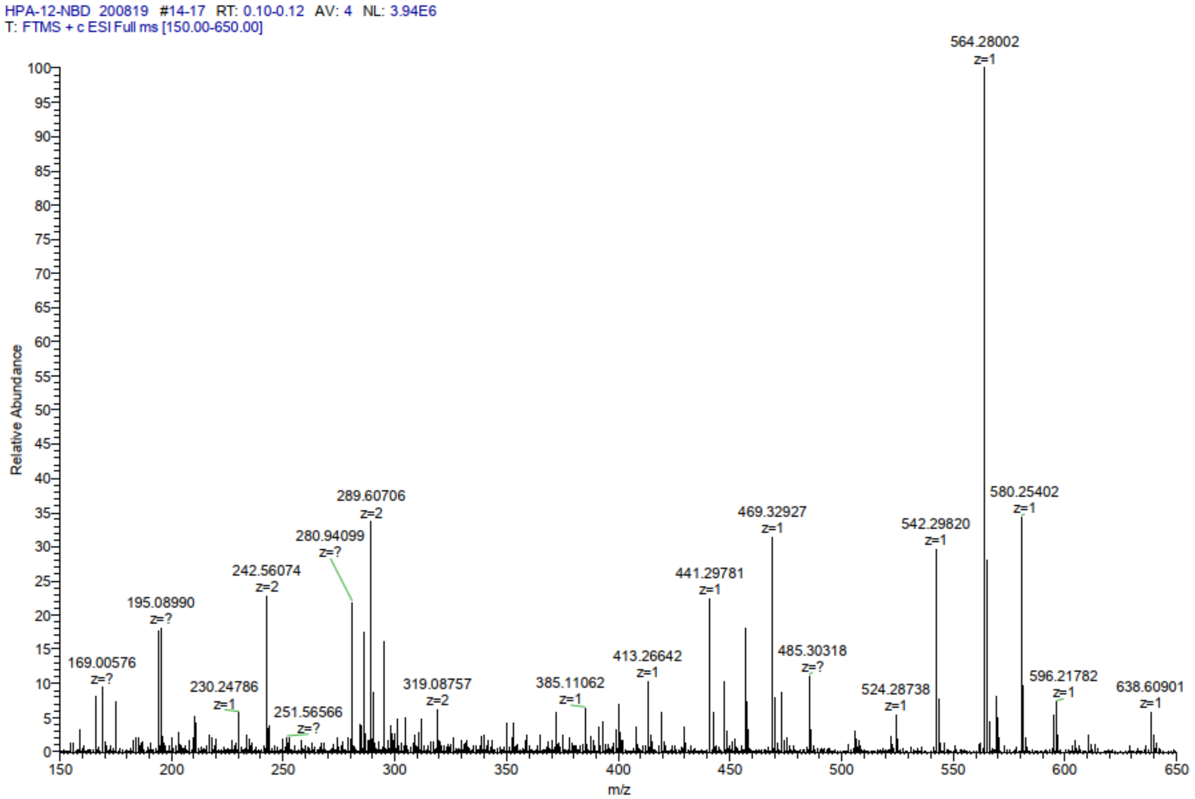

Supplementary Figure 5. HPA-12-NBD HRMS experiment

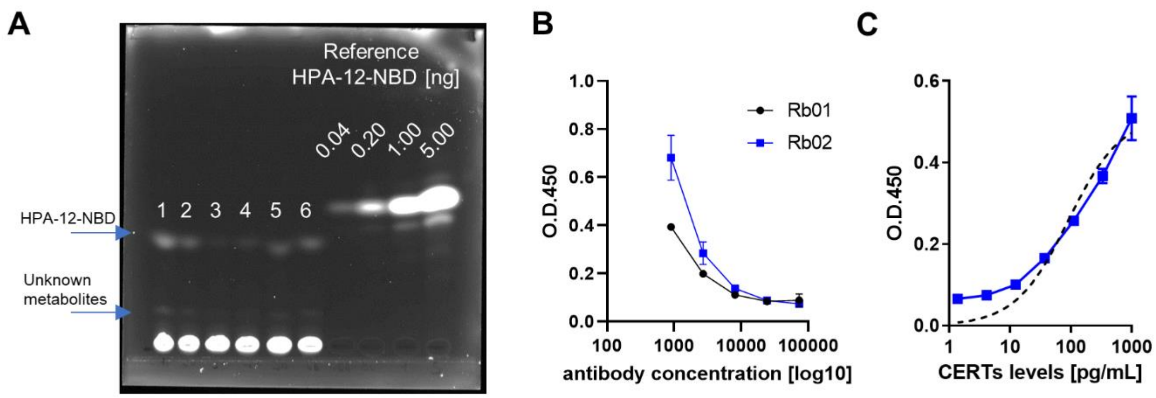

Supplementary Figure 6. HPA-12-NDB separation by TLC and graphs of standard curves validating anti-CERTs antibodies Rb01 and Rb02. A) Image of HPA-12-NBD extracted in chloroform/methanol solution and eluted through silica gel with organic solvent. Pure HPA-12-NBD was used as reference. (1 and 6 = FAD; 2 and 4 = control mice which recieved IV injected; 3 = FAD subcutaneous injection; 6 = FAD intraperitoneal injection). B) Curve representing dilution curve of Rb01 and Rb02 detecting 25 ng of recombinant CERT with a indirect ELISA. C) Calibration curve showing detection of recombinant CERT using Rb01 and biotinylated Rb02 (blue). The dotted curve represent the best fit model for CERTs quantification in samples.
